# Current and Emerging Techniques for Diagnosis and MRD Detection in AML: A Comprehensive Narrative Review

**DOI:** 10.3390/cancers15051362

**Published:** 2023-02-21

**Authors:** Alexandra Teixeira, Luís Carreira, Sara Abalde-Cela, Belém Sampaio-Marques, Anabela C. Areias, Paula Ludovico, Lorena Diéguez

**Affiliations:** 1 International Iberian Nanotechnology Laboratory (INL), Avda Mestre José Veiga, 4715-310 Braga, Portugal; 2 Life and Health Sciences Research Institute (ICVS), School of Health Sciences, University of Minho, 4710-057 Braga, Portugal; 3 ICVS/3B’s–PT Government Associate Laboratory, 4710-057 Braga, Portugal

**Keywords:** acute myeloid leukemia (AML), measurable residual disease (MRD), microfluidics, surface-enhanced Raman scattering (SERS)

## Abstract

**Simple Summary:**

Acute myeloid leukemia is the most common type of leukemia in adults. It is frequently associated with a limited response to conventional therapies that have a high recurrence and mortality rate in the elderly population. Even patients with clinical remission after initial treatment eventually relapse due to measurable residual disease. An earlier and more accurate diagnosis of this condition, using tools with higher sensitivity and specificity, would allow for a more reliable prognosis of patients, leading to more favorable outcomes.

**Abstract:**

Acute myeloid leukemia (AML) comprises a group of hematologic neoplasms characterized by abnormal differentiation and proliferation of myeloid progenitor cells. AML is associated with poor outcome due to the lack of efficient therapies and early diagnostic tools. The current gold standard diagnostic tools are based on bone marrow biopsy. These biopsies, apart from being very invasive, painful, and costly, have low sensitivity. Despite the progress uncovering the molecular pathogenesis of AML, the development of novel detection strategies is still poorly explored. This is particularly important for patients that check the criteria for complete remission after treatment, since they can relapse through the persistence of some leukemic stem cells. This condition, recently named as measurable residual disease (MRD), has severe consequences for disease progression. Hence, an early and accurate diagnosis of MRD would allow an appropriate therapy to be tailored, improving a patient’s prognosis. Many novel techniques with high potential in disease prevention and early detection are being explored. Among them, microfluidics has flourished in recent years due to its ability at processing complex samples as well as its demonstrated capacity to isolate rare cells from biological fluids. In parallel, surface-enhanced Raman scattering (SERS) spectroscopy has shown outstanding sensitivity and capability for multiplex quantitative detection of disease biomarkers. Together, these technologies can allow early and cost-effective disease detection as well as contribute to monitoring the efficiency of treatments. In this review, we aim to provide a comprehensive overview of AML disease, the conventional techniques currently used for its diagnosis, classification (recently updated in September 2022), and treatment selection, and we also aim to present how novel technologies can be applied to improve the detection and monitoring of MRD.

## 1. Introduction

Hematopoiesis is a process that promotes the formation, development, and maturation of all blood constituents from hematopoietic stem cells (HSCs) [1]. HSCs are multipotent cells that can self-renew and have the capacity to regenerate all the different cell types of the hematopoietic system, including the formation of erythroid cells, platelets, neutrophils, monocytes/macrophages, basophils, eosinophils, and lymphocytes [2,3]. These cells have a key role in the continuous replenishment of blood cells throughout life [2]. However, several intrinsic and extrinsic factors might have an impact on hematopoiesis [3,4]. Disturbance in the ability of the HSCs to proliferate and differentiate in the different blood cells can cause an accumulation of immature blasts and hematopoiesis failure, consequently leading to the development of distinct hematopoietic disorders, such as leukemia.

Leukemia can be grouped according to the affected cell lineage and the proliferation rate of immature cells. As such, leukemia can affect myeloblasts (myeloid) or lymphocytic precursors (lymphoid), and can be classified as acute (fast proliferation) or chronic (slow proliferation) [5]. Consequently, leukemia is mainly divided into four types: acute myeloid leukemia (AML), chronic myeloid leukemia (CML), acute lymphoid leukemia (ALL), and chronic lymphoid leukemia (CLL) [6]. Depending on the type of leukemia, patients undergo tailored chemotherapy until they clinically reach complete remission (CR). However, after achieving CR and despite having no symptoms or signs of the disease, some patients may present small undetectable numbers of leukemic stem cells (LSCs) in the bone marrow [7]. This condition is named minimal, or, more recently, measurable residual disease (MRD), and it constitutes the major cause of leukemia relapse. Until very recently, none of the conventional diagnostic tests used was sensitive enough to assess MRD in the early stages. In the following sections, we provide an overview on AML and the current tools used in the clinical setting for their diagnosis, monitoring, and therapeutics, and then focus on the new technologies and their potential to assess MRD.

## 2. Acute Myeloid Leukemia

AML is a heterogeneous group of hematopoietic neoplasms and the most common type of acute leukemia in older individuals [8,9]. AML incidence rises with age, increasing from ~1.3 per 100,000 of the population in those less than 65 years old to 12.2 cases per 100,000 of the population in those over 65 years of age, and with a median age of diagnosis of 68 years old [10]. Prognosis also changes with age, younger individuals having a more favorable prognosis. In the majority of cases, the disease appears as a de novo malignancy in healthy individuals, but patients with underlying hematological disorders or prior chemotherapy are at higher risk of developing the disease [10]. The estimated 5-year overall survival (OS) for older patients, those with secondary AML, or those with relapsed disease is an alarming 5–10%. 

The appearance and even the accumulation of mutations in HSCs and normal dividing cells are often associated with aging [11]. A common age-related condition characterized by the acquisition of somatic mutations in leukemia-associated myeloid malignancy driver genes is clonal hematopoiesis of undetermined potential (CHIP) [12,13]. However, these mutations must be present at a variable allele frequency ≥2% in healthy individuals non-diagnosed with myeloid neoplasm or other obvious hematologic conditions [14]. Taking this into account, CHIP is considered a potential pre-stage of leukemia, associated with a 0.5–1.0% risk (per year) for developing the hematologic disease [14,15,16]. 

AML disease arises from continuous genetic and epigenetic alterations in HSCs and progenitor cells [17,18]. It is known that somatic mutations in genes codifying epigenetic modifiers as *NPM1, DNMT3A, IDH1/2*, and *TET2* are accumulated with age and/or during AML progression in HSCs [17,19,20,21]. Shlush et al. found that there is a potential order for a mutation to happen, therefore mutations in *DNMT3A* and *IDH2* occur in pre-leukemic HSCs, and mutations on *NPM1c* and *FLT3* [21,22,23] and *NRAS* and *RUNX1* [24] appear later in a leukemic state. In addition, even in cases of absence of any large chromosomal abnormality, genetic mutations which are identified in more than 97% of the cases are involved in the development of AML [10]. Furthermore, emerging evidence suggests that LSCs induce molecular changes in the distinct hematopoietic and non-hematopoietic cell populations in the bone marrow (BM) niche [25]. The latter happens via exosome secretion, facilitating the development of a malignant microenvironment while disrupting normal hematopoiesis [26]. However, the conundrum of ‘the chicken or the egg’ of whether the niche favors some mutations or whether the mutations precede alterations in the niche remains an open question. It is possible that these two events are concurrent, where either niche alterations are genotoxic and oncogenic and/or that malignant cells might transform the normal niche to promote their survival and expansion.

**Figure 1:**
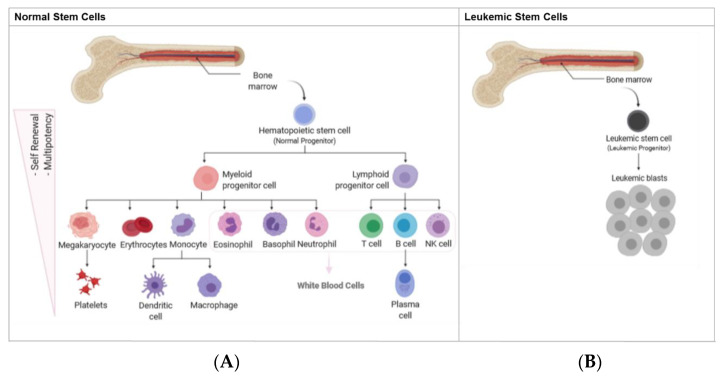
Scheme summarizing the development of leukemia. Representation of normal hematopoietic development (**A**) versus leukemic development and (**B**) when normal hematopoietic development is initiated in the bone marrow by repopulation of hematopoietic stem cells (HSCs) (**A**). Formation of leukemic blasts and leukemia stem cells (LSC) and their accumulation in the bone marrow (**B**).

As a result of the dysregulated differentiation of normal blood cells, an accumulation of immature myeloid progenitor cells is observed not only in the BM (Figure 1), but also in the peripheral blood (PB). Consequently, the ability of the BM to produce normal white and red blood cells and platelets is reduced [25,27,28]. Occasionally, clonal blasts can be found in other organs, such as the liver, spleen, and lymph nodes, resulting in anemia, bleeding, or organ infiltration and, ultimately, in hematopoietic failure [29,30,31]. Finally, due to the highly heterogeneous genetic landscape of LSCs, early detection and personalized treatment constitute the biggest challenges in the field of AML. Furthermore, expanding the knowledge of the genetic landscape might promote a better understanding of therapeutic fragilities in AML, contributing to the definition of new potential targets, the development of new therapies, and the improvement of current strategies [32].

### 2.1. Diagnosis

At the initial stages of the disease, PB analysis showing a high number of abnormal white blood cells (WBC), or even a very low blood count, can indicate the presence of AML. Hence, the confirmation of the AML diagnosis is performed by further morphologic and cytogenetic assessment in BM aspirates [6,33]. The immunophenotyping by multiparameter flow cytometry (MFC) is conventionally used to accurately diagnose AML based on the identification of intracellular and extracellular markers (e.g., CD13, CD33 and CD34). On the other hand, the conventional cytogenetic analysis is mandatory in the assessment of AML disease. An alternative to detect specific abnormalities (*RUNX1::RUNX1T1, CBFB::MYH11, KMT2A (MLL)* and *MECOM (EVI1)* gene fusions or myelodysplasia-related chromosome abnormalities) is fluorescence in situ hybridization. Beyond these technical approaches, different molecular genetic tests have been used to screen all AML characteristic genetic abnormalities, and the tests are needed for differential diagnosis and targeted treatments. 

The final diagnosis and monitoring of AML always involves a BM tissue biopsy (BM trephine biopsy) or a BM biopsy. Those are invasive, painful, and create discomfort and harm for leukemia patients [34]. Therefore, less invasive procedures are needed while ensuring an accurate diagnosis revealing more detailed information about the disease progression.

One of the less invasive procedures that have been extensively studied and applied as a complementary method is the PB biopsy, also known as liquid biopsy [6]. This is currently a complementary technique to the gold standard method for the detection of leukemia or MRD because of a smaller number of LSCs in the PB when compared with BM aspirates [34].

### 2.2. Classification

The classification of hematological malignancies has advanced tremendously since the 1970s. The classification of AML is based on two main systems: the French-American-British (FAB) and the World Health Organization (WHO) (Table 1) [24,35,36,37,38,39]. The FAB classification was introduced in 1976 and it is based on the morphology and cytochemistry of blasts, the degree of myeloid or monocytic differentiation, and their degree of maturation [35,36,40]. As such, this classification recognizes eight distinct subtypes of AML, from M0 to M7 [35,40]. This system has been widely used for the classification of hematological disorders in the last decade since characterization of the blasts is easily accessible and low cost. However, with recent advances in the genetic and molecular characteristics of AML, this classification is now becoming incomplete and outdated. Consequently, the WHO system has been increasingly used to classify AML. This system was firstly introduced in 1999 [17,41] and reviewed in 2016 [42], combining the previous features from the FAB classification with genetic data (chromosomal and molecular), biological data, and clinical data [42]. More recently, in September 2022, new updates about AML classification were published [39]. This updated version of the classification provides a more genetically defined classification as it retains most of the previously defined AML types (with recurrent genetic abnormalities) and includes other genetically-related entities to define AML and changes in the blast thresholds. In summary, this revision of the WHO classification (completed by leading experts), maintains the blast threshold at 20% for the diagnosis of the majority of AML subtypes, but decreases it to ≥10% blasts in BM or blood in the presence of recurrent genetic abnormalities (except for AML with t(9;22)(q34.1;q11.2)/*BCR::ABL1)* (Table 1) [24,39]. The case of AML with *BCR::ABL1* still requires ≥20% blasts, due to avoiding overlap with chronic myeloid leukemia in the accelerated phase [24,39].

In addition, some predisposing features (therapy-related, prior myelodysplastic syndrome (MDS) or MDS/myeloproliferative neoplasm (MPN), and germline predisposition) designed as qualifiers of the primary diagnosis have also been added. The WHO system provides a better characterization of AML patients, offering clear advantages for treatment personalization [17,41]. 

### 2.3. AML Pathogenesis and Prognosis

In the new diagnosis of AML, a high percentage (50–60%) of cytogenetic abnormalities are found [17]. These abnormalities are usually associated with non-random chromosomal translocations that result in gene arrangements implicated in a modification in the locus encoding for a transcriptional activator, leading to the expression of a fusion protein [43]. These fusion proteins promote changes in the expression of target genes responsible for myeloid development maturation and proliferation, an abnormality that potentiates AML transformation [44]. As a result, AML is constituted by a heterogeneous population of malignant cells. The different AML clones have distinct cytogenetic alterations and mutations that produce considerable genetic complexity. Taking this into account, a risk classification system in AML patients at diagnosis was also defined. This system was also updated this year (September, 2022), but maintained the division into three risk categories: favorable, intermediate, and adverse [24]. However, it is important to note that after the recent revision and refinement, in addition to the baseline genetic abnormalities, the response to initial therapy and early MRD testing are now considered factors that contribute to the patient’s risk classification [24]. Thus, AML patients with a favorable prognosis present translocations of t (8; 21) (q22;q22.1)/*RUNX1*::*RUNX1T1* with an occurrence of 10%; inversions of inv. (16) (p13.1q22) with an occurrence of 5% [17,42,45]; and mutated *NPM1* without *FLT3*-internal tandem duplication (ITD) and in-frame mutations affecting the bZIP region of *CEBPA* [24].

The intermediate risk group includes all AML cases with *FLT3*-ITD; mutated *NPM1* with *FLT3*-ITD; wild-type *NPM1* with *FLT3*-ITD; with translocations: t(9;11)(p21.3;q23.3) involving the *MLLT3*::*KMT2A* genes, and all the cytogenetic and/or molecular abnormalities not classified as favorable or adverse [24].

Finally, the adverse-risk group now includes recurring cytogenetic abnormalities, such as the translocations: t(3q26.2;v) involving the *MECOM* gene; t(8;16)(p11;p13) associated with the *KAT6A::CREBBP* gene fusion; t(6;9)(p23;q34.1)/*DEK::NUP214;* t(v;11q23.3)/*KMT2A*-rearranged; t(9;22)(q34.1;q11.2) involving *BCR::ABL1* genes; t(3;3)(q21.3;q26.2) involving the *GATA2, MECOM(EVI1)* genes or inversions: inv(3)(q21.3q26.2); −5 or del(5q); −7; −17/abn(17p). AML patients that present hyperdiploid karyotypes with multiple trisomies and complex karyotypes or monosomal karyotypes are also included in this group. Additionally, mutated *ASXL1, BCOR, EZH2, RUNX1, SF3B1, SRSF2, STAG2, U2AF1,* or *ZRSR2* and mutated *TP53* are also classified as adverse risk [24]; and if adverse-risk cytogenetic abnormalities are present in *NPM1*-mutated AML, they are also classified as adverse risk [24]. Patients included in the adverse risk group (~40%) have reduced rates of CR being prone to develop recurrence [23,46]. 

### 2.4. Therapy and Outcomes

The current AML intensive treatment protocols are the same that were established more than 50 years ago. The remission-inducing chemotherapy usually consists of a combination of high doses of two cytotoxic drugs, cytarabine and anthracycline, with or without a purine analog [24,47,48,49,50]. After the induction therapy to clear LSCs and consequently induce CR, patients follow consolidation chemotherapy regimens (consisting of an intermediate dose of cytarabine) to deepen remission and maximize response [24,51]. In some cases, maintenance therapy is also applied to patients that have achieved remission, with the main goal of decreasing the risk of relapse [24]. In other cases, such as patients classified with adverse risk, allogeneic hematopoietic stem cell transplantation (alloSCT) is also recommended, but it is dependent on the risk of relapse (>35% to 40%), age (<60 years), and other comorbidities [24,52]. Although conventional therapy has better results in younger patients, this approach presents poor results in elderly individuals or in patients that present some comorbidities [53]. This is due to the combination of the high toxicity of the drugs and the heterogeneity of the disease [9,54,55,56]. Thus, some alternative treatment options are idarubicin (FLAG-IDA), fludarabine, cytarabine, azacitidine, decitabine, granulocyte colony-stimulating factor, and also mitoxantrone-based cytarabine regimens.

Current research has been devoted to the identification of molecular targets allowing the implementation of personalized treatments. In 2017 and 2018, a handful of new drug formulations were approved by the FDA: new liposomal formulation of cytarabine and daunorubicin (CPX-351), a BCL-2 inhibitor (venetoclax), the IDH inhibitors (ivosidenib and enasidenib), the FLT3 inhibitors (gilteritinib and midostaurin) the anti-CD33 monoclonal antibody gemtuzumab ozogamicin, the hedgehog signaling pathway inhibitor (glasdegib), and the oral HMA CC-486 [17,50,57,58,59,60,61]. Some of these therapeutic options have already demonstrated very good results in clinical trials. For example, CPX-351 treatment improved the 5-year OS by 10% in patients aged 60–75 years, and with newly diagnosed high-risk or secondary AML when compared with conventional chemotherapy (cytarabine for 7 days plus daunorubicin on days 1, 2, and 3 [7 + 3 regimen] and cytarabine for 5 days and daunorubicin on days 1 and 2 for the second induction [5 + 2 regimen]) [62]. The use of a kinase inhibitor, midostaurin, as an adjunct to conventional therapy, daunorubicin and cytarabine (induction therapy), and then to high doses of cytarabine (consolidation therapy) also promoted an increase in overall and event-free survival in AML patients with *FLT3* mutation, and, more precisely, increased the 4-year OS from 44.3% to 51.4% [63]. Currently, midostaurin is often used as first-line therapy in AML patients with FLT3 mutation [63]. 

Despite these advances, the choice of the therapeutic regime (combination of drugs on doublet and triplet regimens) has always been based on the patient’s eligibility or ineligibility to undergo intensive chemotherapy [8,17]. It is important to note that there are still no generally accepted or validated criteria for considering a patient ineligible for intensive therapy. However, in the context of clinical trials, there are some criteria to consider a patient not suitable, which can be used as a guideline in routine practice [24]. According to the 2022 revision completed by Dohner et al., patients not suitable for intense therapy are (1) patients >75 years old (cannot be an absolute criterion, because AML patients with a more favorable disease and without relevant comorbidities can be submitted to intensive chemotherapy); or (2) Eastern Cooperative Oncology Group (ECOG) performance status >2 and/or age-related comorbidities, such as severe cardiac disorder, severe pulmonary disorder, creatinine clearance <45 mL/min, a hepatic disorder with total bilirubin >1.5 times, or any other comorbidity that the physician assesses to be incompatible with intensive chemotherapy [24]. Thus, for patients who are not candidates for intensive remission-induction therapy, venetoclax plus either hypomethylating agents or low doses of cytarabine have emerged as an effective treatment option based on the achievement of a high and rapid rate of response [24,64,65]. Finally, for patients that do not want any treatment or cannot tolerate any type of anti-leukemic therapy, the best support care is proposed.

More recently, immune therapies based on antibody therapies have revolutionized the treatment of leukemia. This includes chimeric antigen receptor T cells (CAR T cells), which are T cells taken from the patient and educated in the laboratory to express a specific antigen receptor target or CAR designed against a specific cell-surface antigen. These immunotherapeutic strategies have shown good results in other types of leukemia, particularly CLL and ALL [66,67], showing superior remission rates when compared with conventional therapy regimens. Despite several efforts to achieve similar success of antibody-based or cellular immunotherapies in AML [68], it is still a challenge to identify a good target antigen for this hematologic malignancy. 

In conclusion, due to the heterogeneous nature of AML, there is a pressing need for more fundamental research and biomarker identification to optimize and personalize AML therapies. Advances in this field will have the potential to improve quality of life, increase the time of at-home care, and reduce early organ damage and mortality while reducing the clinical, emotional, psychosocial, and economic burden associated with current intensive therapies.

## 3. Measurable Residual Disease (MRD)

**Figure 2:**
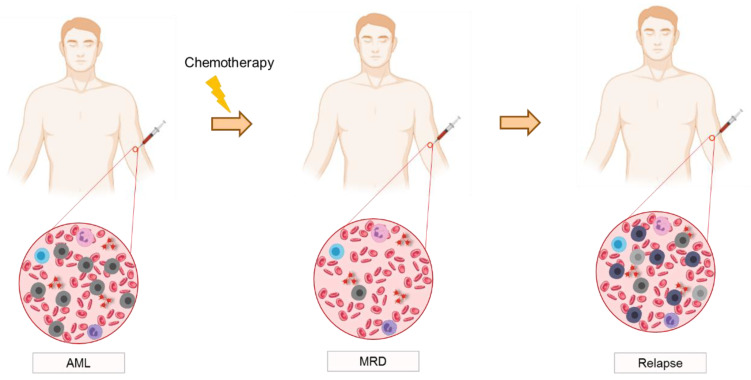
The sequence of events in acute myeloid leukemia from diagnosis to relapse: AML disease diagnosis followed by chemotherapy, MRD detection and relapse.

After the first induction of chemotherapy, CR rates reach approximately 80% and the disease burden is reduced by 99.9%. However, some residual LSCs (up to 10^10^ to 10^12^ cells) can remain, causing almost every patient to relapse if no consolidation therapy is provided [69,70]. Even when consolidation therapy is performed to eradicate any persistent LSCs, above 50% of patients will still relapse or develop refractory disease [71,72,73] (Figure 2). This is caused by the persistence of LSCc not detected by conventional technologies, and constitutes the termed minimal residual disease. However, more recently (in 2018), this nomenclature has been replaced by measurable disease (MRD) [74]. The term “measurable” has been proposed to indicate contexts where levels of LSCs are detectable by modern technologies that already have a higher sensitivity [70,75,76]. 

MRD monitoring is performed routinely in clinical practice since it is known that the presence of MRD is a strong prognostic indicator of relapse risk. Besides, MRD can also have implications in the planning and personalization of treatment, when assessed in conjunction with other well-established clinical, cytogenetic, and molecular data [77,78]. Furthermore, it has been shown that when MRD detection is successfully performed at an early stage, it results in improved prognosis, disease management, and patient outcome [79]. 

Given the importance of efficient and sensitive detection of MRD, which to date remains challenging, a large body of research has concentrated on developing new precise and sensitive detection strategies. Due to AML and the short period between CR and relapse, finding particular genetic mutations or aberrant protein expression patterns constitutes a major challenge [80,81,82,83]. Additionally, the challenge of detecting a MRD condition is due to the fact that the number of leukemia cells is very low compared to other blood cells; i.e., in ratios of 1:10^4^ to 1:10^6^ LSCs to WBCs, which makes this type of evaluation an extremely difficult task [75,84,85,86,87]. Thus, powerful and highly sensitive techniques are required to perform the molecular MRD assessment, reaching a limit of detection of 10^−3^ or lower, and PB and BM may be used [74,88]. 

### The Emerging Role of MRD in the Therapeutic Scenario

Recently, the research community has demonstrated the relevance of MRD assessment towards guiding and personalizing therapy in leukemia patients [89]. The results of studies and clinical trials carried out in this field point towards two main MRD-driven patient management strategies: (i) the use of MRD in the pre-transplant scenario (induction and before transplantation), helping to select the most appropriate consolidation therapy and risk stratification; and (ii) the evaluation of MRD in the post-transplant setting, such as taper off maintenance therapy [89,90,91,92,93]. Thus, the stronger added value of MRD is based on its use as a (a) monitoring tool to detect imminent disease relapse, (b) a prognostic biomarker to allow the refinement of post-remission relapse risk assessment, and also (c) as a potential surrogate endpoint (still under exploration) [70,74,94]. In agreement, the recent *‘Diagnosis and management of AML in adults: 2022 recommendations from an international expert panel on behalf of the ELN’,* recommends MRD assessment only after two cycles of induction/consolidation treatment, at the end of treatment, and during follow-up (every 3 months for up to 24 months) [24]. This recommendation validates not only the more frequent assessment of MRD at different and specific time points during treatment, but also the application of MRD as an independent biomarker to predict outcome, and an asset for detailed patient monitoring [24,95,96]. 

Although MRD negativity has been best stablished as a potential surrogate for clinical benefit in ALL, APL, and chronic lymphocytic leukemia, for AML this is still unclear due to the heterogeneity of the disease and the complexity of the MRD assessment [89,95]. In AML, MRD assessment is relevant for understanding the efficiency of induction chemotherapy and the intensity of the conditioning regimen; determination of the optimal type of post-remission therapy, such as hematopoietic stem cell transplantation in patients at high risk of relapse or maintenance therapy with hypomethylating agents; and choice of immunosuppressive agents, among others [94,96]. 

Currently, the use of MRD analysis to guide treatment decisions in AML patients is still not widely accepted, despite being a promising strategy. Nevertheless, many clinical centers have already started to guide treatment based on MRD, especially in the early stages of disease, given its strong clinical relevance and its potential to aid therapeutic decisions [95]. However, it is important to mention that most of the information until now is derived from clinical studies in patients submitted to intensive treatment. In the context of less intensive treatments (hypomethylating agents treatment or targeted therapies), the investigation about the predictive value of MRD assessment is still less extensive [95]. 

For example, MRD detection on AML patients with CR guides the early decision to proceed with an alloSCT. Post-transplantation monitoring of MRD could also guide the prescription of immune-suppressive drugs or more intensified chemotherapy regimens or with hypomethylating agents plus venetoclax; targeted therapy combinations when indicated, usually taking into account specific molecules abnormalities (FLT3 or IDH inhibitors); and antibody therapies [97]. 

While having already demonstrated the high relevance of MRD detection, one of the challenges is still to be able to detect this condition, particularly in the early stages. Thus, it is crucial to use reliable tools, and with high sensitivity.

## 4. Traditional Techniques for Diagnosis and Monitoring of MRD

The European LeukemiaNet MRD Working Party has released comprehensive, consensus recommendations for the use of appropriate techniques having taken into consideration their technical limitations [98]. Well-established techniques, such as reverse transcription polymerase chain reaction (RT-PCR) and multiparameter flow cytometry (MFC), are recommended by the European Leukemia Network AML Measurable Residual Disease Expert Committee to detect MRD in AML. Additionally, other strategies have also been applied as well as next-generation sequencing (NGS) [75]. All these techniques present advantages and disadvantages regarding levels of sensitivity, applicability to the various heterogeneous types of AML, and costs and level of technical expertise needed to process the data. It is worth noting that an ideal MRD detection method should discriminate different cellular populations that could or not cause relapse. Except for the detection of acute promyelocytic leukemia by PCR, current detection methods are not sensitive enough to detect MRD in a regime with very low residual LSCs [84,99]. 

### 4.1. Multiparametric Flow Cytometry (MFC)

MFC is one of the most common techniques used for the diagnosis, classification, and prognosis of AML disease. This technique identifies a signature of aberrant expression of antigens present on AML cells, through specific panels of fluorochrome-labeled monoclonal antibodies [100]. Recent advances, based on the development of multicolor panels (>8 colors), brought the levels of sensitivity of cytometric analysis closer to those obtained by molecular biology techniques [75,84]. Thus, MRD analysis based on MCF presents moderate sensitivity, with a LOD of 1:10^−3^ up to 1:10^−4^ (1 leukemic cell in 10,000 to 10,000 WBCs), which is dependent on the number of cells analyzed, the gating strategy, and the number of antibody markers used [24,70,98]. The main advantage of MCF over other techniques is the ability to characterize millions of cells in a short period of time, while distinguishing viable cells from debris and dead cells [101,102,103]. Furthermore, it has the advantage of being applicable to the vast majority of AML patients, unlike other techniques that focus on the genetic and/or molecular signature [98].

MRD condition can be assessed/monitored in AML patients by MFC, based on leukemia-associated immunophenotypes (LAIPs) [104,105]. Briefly, it is known as LAIPs, the abnormal expression of immunophenotypic markers that provide the distinction of the leukemic cells. Conventionally, for the detection of LAIPs, several markers can be used, including CD34, CD117, CD45, CD33, CD13, CD56, CD7, HLA-DR (for MRD), among others [24]. Much work has been completed to improve this strategy and its application in MRD. In most of the cases, the improvements consist of the combination of several antibodies with different fluorophores (at least eight) to detect more than one LAIP at a time, improving the diagnostic performance [106,107]. 

Several other studies were conducted to understand the ability and efficiency of cytometric analysis to detect MRD. For example, a study with 451 BM samples from adult AML patients assessed the clinical utility of MRD detection, demonstrating that MFC is useful for outcome prediction and guidance of post-remission in AML [108]. 

The biggest drawback of this technique is the requirement of significant technical expertise and the intervention of an interpreting pathologist. The future use of artificial intelligence to reduce potential bias and/or human interpretive errors can bring accurate interpretations of the results. The low sensitivity and the high change of the aberrant immunophenotype during disease progression makes the assessment of MRD more difficult, leading to a high rate of false negatives and limiting the detection of the different aberrant markers.

### 4.2. Molecular-Based Techniques

Molecular techniques are used to assess the presence of MRD by analyzing well-known AML mutations [24,70]. The most common markers used to monitor MRD are *NPM1* insertion, *CFB-MYH11*, *AML1-ETO*, and *PML-RARA* (chimeric fusion genes) [109]. *NPM1* is one of the most prevalent mutations in AML (present in 25–35% of patients) and is reported as a definitive leukemia-founder mutation that has already been validated as an optimal and reliable MRD marker [110,111]. Recent studies are focused on introducing/validating new markers for MRD monitoring; for example, many studies are focused on the relevance of *IDH* mutations [24]. The *IDH1* and *IDH2* mutations area is also prevalent in AML (occur in ~20% of cases) but there are still conflicting data, whether they represent pre-leukemic mutations or dominant clonal mutations, hence their value in monitoring MRD is not yet well established [75,110,112,113]. Moreover, *NPM1* is frequently one of the references for the studies involving *IDH* mutations [114,115]. 

Furthermore, the prognostic significance of combined mutations, such as *NPM1, FLT3, DNMT3A*, and *IDH* is still unclear. However, recent studies state that some combinations may have an impact on the risk of relapse and overall survival of AML patients compared with other combinations [116,117,118]. Thus, in AML disease, and even in the MRD condition, it is crucial to use reliable and sensitive molecular techniques to detect these mutations, even when present in small amounts, in order to have a better understanding of their prognostic value.

#### 4.2.1. Reverse Transcription Polymerase Chain Reaction (RT-PCR)

In AML, the development of RT-PCR has been crucial to detect residual elements conventionally characterized by molecularly cloned chromosome translocations [119,120]. The use of RT-PCR enables the detection of the fusion genes *RUNX1-RUNX1T1* and *CBFB-MYH11* [121] as well as *PML-RARA* [122,123] and mutated-*NPM1* [86,124], important for the sensitive detection and quantification of MRD in AML [75,125]. In fact, in the case of CBF fusion transcripts (*RUNX1-RUNX1T1* and *CBFB-MYH11*), some studies in PB or BM have demonstrated the prognostic value of detection and quantification of MRD at specific time points allowing the identification of patients with a high risk of relapse [121,126]. In addition, several studies have investigated the clinical implications of monitoring *NPM1* and its association with relapse and survival rates [127,128]. In the study performed by Ivey et al., using BM and PB of the AML patients, the presence of *NPM1*-mutated transcripts after the second chemotherapy cycle was associated with a significantly higher relapse risk and poorer survival rates, independent of other known prognostic factors [127]. 

Given the high specificity and sensitivity for LSCs, decreased risk of contamination and better evaluation of RNA quality, the technique of RT-PCR has been widely implemented for routine patient care [70]. However, the detection of MRD based on RT-PCR or PCR is currently limited to around 50% of patients, since not all patients carry the fusion transcript *RUNX1-RUNX1T1* (characteristic of t[8;21]), *CBFB-MYH11* [inv 16] or t[16;16]), or *NPM1* mutations [91]. The optimized RT-PCR assays presents a limit of detection (LOD) of 10^−4^ to 10^−6^, and is therefore more sensitive or equal than other technologies used, such as MFC (range 1:10^−3^ up to 1:10^−4^ ) [75,129,130,131].

As is the case with any other technique, there are some limitations of RT-PCR-based MRD tests, namely their dependence on specific mutations, requiring individual standard reference curves based on serial dilutions of targets, intensive labor, expertise, cost, time-consuming, and computationally demanding work [84,132].

#### 4.2.2. Next-Generation Sequencing (NGS)

Next-generation sequencing (NGS) refers to the deep, high-throughput, in-parallel DNA sequencing technologies that were developed in the decades after 1977, when the Sanger DNA sequencing method was developed. Unlike Sanger sequencing, NGS procedures provide a massive parallel and extremely high-throughput analysis (millions to billions of nucleotides) of multiple samples, bringing much faster results (due to multiplexing), and at a lower cost than individual testing [133]. Therefore, NGS is a tool that easily assesses genomic, transcriptomic, and epigenomic features [134]. More specifically, in the AML context, NGS has extreme relevance for diagnosis, due to the high clonal heterogeneity characteristic of this disease [135]. It provides information about the different AML mutations present in a patient, providing more information and allowing the design of personalized therapies, which will ultimately result in a better prognosis of remission and less cases of relapse [84]. It was already described that for patients in CR, the estimated percentage of the MRD measured by NGS was much greater than of aberrant blasts detected by MFC [136].

This technology has also revealed a set of mutations in rare mutant cells and gene sequences, as well as the discovery of genetic alterations that occur between diagnosis and relapse times [137]. In a study with a cohort of 482 patients, at least one mutation was detected in 430 patients (89.2%) showing the broad clinical application of targeted NGS [138]. Additionally, NGS showed a significant additive prognostic value compared to flow cytometry for the detection of MRD [138].

Recently, Alonso et al. tested a 19-gene AML-targeted NGS in a small cohort of 162 patients and showed that well-defined NGS panels are reliable in guiding clinical decisions by the current standards. Results had a 100% correlation with conventional molecular biology techniques, and all patients were successfully classified according to 2016 WHO classification systems (2016 WHO diagnostic categories, 2017 European LeukemiaNet, and Genomic classification) [139]. NGS was also explored in the context of allogeneic hematopoietic cell transplantation (alloHCT). For example, Thol et al. published a clinical study with a cohort of more than one hundred patients, demonstrating that MRD detection utilizing NGS before alloHCT is highly predictive of relapse and survival, and can therefore improve patient management [140]. It is worth noting that in this study similar results were obtained from both BM and PB samples, demonstrating the usefulness of using less invasive body fluids [140]. Thus, through the molecular characterization of AML cells, NGS promotes a personalized and precise method for the assessment of MRD, since can reach a sensitivity of 10^−4^ to 10^−5^ [95, 132]. However, the widespread use of NGS has been limited due to its high cost, and the expertise required for the data analysis and interpretation [94,141]. Furthermore, other drawbacks of this technique are the non-standardization of the assay, the high variance of sensitivity across different platforms, and a long waiting time for the result analysis.

In summary, MRD has been shown to be an important and independent prognostic factor and predictor of relapse, and also plays a crucial role in the design of personalized treatment strategies. However, when values of cells are lower than 10^−5^, it is generally more challenging to be detectable by the diagnostic procedures used in clinical settings. Thus, the development of the aforementioned techniques enables a more reliable and satisfactory detection of MRD and CR as well as the stratification of patients into different subtypes. Still, the sensitivity of some of these techniques is far from ideal, and their implementation in clinical routine is still pending. Despite the technological challenges, it remains crucial to develop new technologies for MRD detection and quantification that are more affordable, faster, and offer higher accuracy and sensitivity.

## 5. Microfluidics for MRD Detection in AML Disease

### 5.1. Microfluidics in MRD Condition

The major limitation of current MRD detection techniques is the inability to detect very low amounts of LSCs, considered rare tumor cells and named circulating leukemic cells (CLCs). These type of cells are present in body fluids, such as BM or PB. Hence, the pressing need of developing new tools with high sensitivity that could be easily implemented in clinical practice. Microfluidic systems are able to process fluids at high throughputs in microchannels. Due to the channel dimensions, in the order of 10 s to 100 s of micrometers, and to the laminar flow conditions, these devices exhibit many advantages to manipulate cells from a complex sample. Microfluidic devices also display a very high surface-to-volume ratio, holding extremely low amounts of fluids (10^−9^ to 10^−18^ L) [142]. The substantial reduction of the scale of the experiments offers unique advantages compared to more conventional analytic approaches, such as reducing the necessary amounts of samples and reagents. Microfluidics is also compatible with multiplexing and automation, and allows quick and efficient cell separation and detection at high resolution, high sensitivity, and low costs [142,143].

Currently, there are reports of many microfluidic devices that offer the capacity to selectively isolate cells from a mixture using several methodologies based on different cell characteristics, such as size [144], deformability [145,146,147], density [148], electrical [149] and magnetic properties [150,151], and surface charge [152,153]. Microfluidic strategies for cell isolation also exploit immunoaffinity, either for negative [154,155] or positive [147,156,157] enrichment of cells in body fluids, or using a combination of both [158]. These assays are not only very efficient but also maintain cell integrity, which is crucial for downstream analyses. Negative enrichment consists of using immunoseparation methods to target specific antigens on the membrane of unwanted cells, for example targeting the CD45 surface marker for the depletion of WBCs [159]. This method allows an unbiased separation of target cells, but it results in lower purity when compared with positive enrichment approaches [160]. The CellSearch^®^ system is an example of a positive enrichment technique that has obtained FDA clearance for the separation of Circulating Tumor Cells (CTCs) from the blood of metastatic patients. In this case, magnetic nanoparticles immunoconjugated to target EpCAM are used to separate the cells of interest from the whole blood sample. Although positive selection promotes greater purity of CTCs, this strategy fails to isolate cells with low expression or negative expression of the surface-marker used [161,162].

Similarly to CTCs, microfluidics can be used to isolate CLCs from body fluids. The application of microfluidics in AML has been explored in many other ways, including a very interesting work for single-cell DNA sequence [163]. In this review, we have focused on microfluidics as a tool to isolate CLCs in order to demonstrate the high relevance of this technique in the MRD detection as a less invasive tool for patient diagnosis and monitoring. Enriching and isolating CLCs would not only provide a way to better detect and monitor MRD (where the number of LSCs is very low), but it would also be invaluable for early diagnosis and accurate patient stratification.

### 5.2. Microfluidics for Isolation of Circulating Leukemic Blasts (CLCs)

Recent studies have shown the capacity of microfluidics to isolate CLCs, which can be used as biomarkers to determine the occurrence of MRD in AML patients [79]. However, the isolation of CLCs can be more complicated than that of CTCs, since they do not have any ubiquitous characteristic that can aid their identification. It is important to emphasize that CLCs and CTCs differ in both, morphological and biological aspects. The most evident parameter is the size, while CTCs have bigger sizes (~14–25 μm), CLCs are a very similar size to WBCs (~7–15 μm) [164,165]. Therefore, in the case of CLCs, it is not useful to use filtration systems or other types of size-based assays.

**Figure 3:**
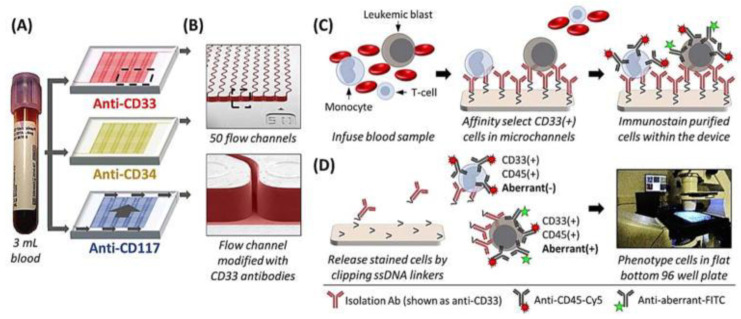
Whole blood is processed through three microfluidic devices modified with mAbs specific for CD33 (red), CD34 (yellow), and CD117 (blue) expressing cells. (**A**) SEMs of the sinusoidal channel array (50 channels in the array) and the entrance of the single channel that addresses all sinusoidal channels. (**B**) Schematic of the affinity isolation assay. (**C**) Selected cells are then immunostained against CD45 and the aberrant marker, and selected cells are released from the capture surface and carried hydrodynamically into flat-bottomed wells, where the cells are imaged. (**D**) CLCs are identified by positive aberrant staining (aberrant (+)) and positive CD45 and DAPI staining, whereas other blood components only show CD45 and DAPI staining (aberrant(−)). Reproduced with permission from Jackson et al. [79].

Another very common technique described to isolate CTCs is positive immunoisolation. The antibodies most commonly used for this purpose are the ones that recognize epithelial cell adhesion molecule (EpCAM). This approach renders high levels of purity because this marker is highly expressed in cancer cells from solid tumors, while WBCs do not express this glycoprotein. On the other hand, antibodies that are used in the context of AML to promote the enrichment of CLCs can also be expressed by blood cells, so the number of interfering cells would be higher, meaning low purity outcomes. Therefore, to overcome these obstacles, an additional step is necessary to distinguish cancer cells from healthy blood cells, using specific markers of aberrant leukemic cells. In this sense, and to detect MRD in patients with AML, Jackson et al., used a microfluidic assay to target several antigens, including CD33, CD34, and CD177 [79]. The antibodies used to recognize these antigens cover about 70% to 90% of the AML patient population. They also included CD7 and CD56 to detect aberrant markers and consequently identify the CLCs fraction of the total of cells isolated (Figure 3). The study also demonstrated that this microfluidic assay is able to detect relapse two months earlier when compared with the technologies currently used––PCR or flow cytometry analysis of a BM biopsy [79].

**Figure 4:**
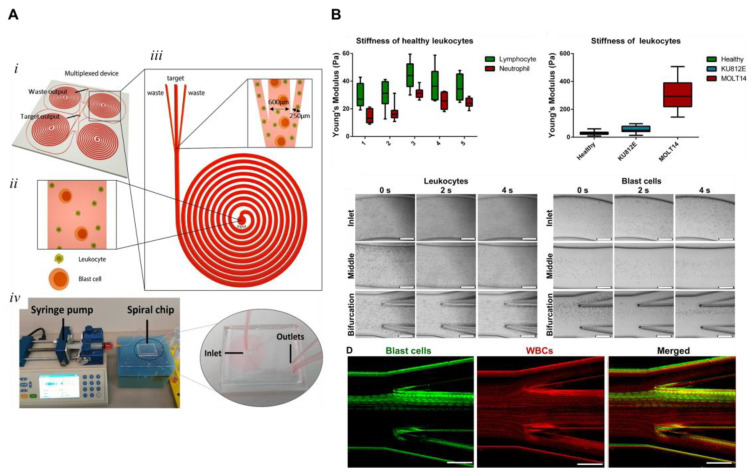
Overview of blast cell isolation using inertial microfluidic chip, blast cell biochip (BCB). (**A**) 3D overview of BCB: (i.) distribution of unselected samples at the entry position; (ii.) distribution of samples at the bifurcation point; (iii.) biochip incorporated into the screening system; and (iv.) enrichment procedure of leukemic blast cells using BCB. (**B**) From top to bottom, results obtained with healthy leukocytes for cell stiffness measurements: cell stiffness of blast cell types compared to healthy leukocytes; characterization of the distribution of healthy leukocytes and target cells under optimal flow rate; and, finally, frames captured with a high-speed camera demonstrating the focused flows of target cells among the other blood cells. Reproduced with permission from Khoo et al. [34].

Recently, an inertial microfluidic chip based on cell stiffness (LSCs are stiffer than the non-target cells) and differential cell size to detect rare leukemic cells was developed by Khoo and collaborators (Figure 4) [34]. For the optimization and validation of the system to detect low amounts of cells in blood, the authors started using samples spiked with LSCs (cell count < 5%) showing a detection limit of 5 LSCs among 10^6^ leukocytes. This efficiency exceeds the minimum detection rate obtained by the methods currently used. After preliminary validations with cell lines, the study used clinical blood samples (including ALL, MDS for various subtypes of AML patients) demonstrating that the system had the ability to promote rapid enrichment of blasts, although the blood samples had to be pre-processed before being run in the system to remove red blood cells [34]. After isolation, the morphology and expression of the recovered cells was identified by cytopathological and/or immunocytochemistry analysis [34]. This procedure allows the potential detection of low amounts of blasts and, subsequently, has added value in the detection of MRD.

In a recent work, Lai et al. also developed a non-invasive strategy for LSCs capture designed LSC-Chip (Leukemic Stem Cell Specific Capture Chip) [166]. This strategy consists of microfluidic herringbone chips with a CD34-antibody-modified surface. To bind the antibody to the surface of the chips, they used a reversible disulfide strategy to promote further analysis, including scRNA-Seq [166]. The main objective of the herringbone structures is to ‘force’ cells to travel through streamlines, and to interact more frequently with the antibody-modified surface, with the primary aim of improving target cell capture. The capture efficiency of the chip was tested using different cell lines such as KG-1a (CD34+) and HCT116 (CD34-). The best results obtained reached a value of 84.55% using KG-1a cells in PBS and 77.75% using the same cells spiked in whole blood, and both used a flow rate of 1.5 mL/h. The LSCs chips were validated using PB samples (non-invasive sampling) from 36 AML patients diagnosed as CR and non-remission, and 10 healthy donors [166]. The results achieved demonstrated the potential of the application of the LSCs-Chip for remission status monitoring in AML.

A novel approach aimed at the isolation and detection of AML cells, more precisely drug-resistant ones, has recently been implemented based on the combination of superparamagnetic nanomaterials and microfluidics [167]. The magnetic capture efficiency of the cells of interest using the microfluidic device, which in this case were HL-60 cells that overexpress CXC chemokine receptor 4 (CXCR4), was 82.92% [167].

All these studies have demonstrated the relevance and potential of using microfluidics to separate/isolate cells of interest more efficiently, with lower sample volume consumption and in a shorter time compared to traditional methods.

## 6. Emerging Technologies for the Diagnosis of MRD

### 6.1. Digital PCR (dPCR)

The inclusion of new techniques with the ability to identify disease-related mutations present at very low frequencies is crucial for the assessment of the MRD evaluation in the clinical and research stages. Digital PCR (dPCR) is a high-performance technology that produces an absolute quantification. It is based on the amplification of target genes without a standard reference curve, unlike its conventional version, RT-PCR [168,169,170]. Due to its advantages when compared with conventional PCR, dPCR is starting to be explored as a replacement of the outdated version in numerous applications, and AML is no exception. Compared with NGS, it allows less multiplexing, but it is cheaper and presents a good sensitivity. In this context, Hindson et al. compared the microfluidic-based system (dPCR) with traditional Taqman probe (or similar) based quantitative PCR systems, which resulted in even greater sensitivity (up to 10 times), accuracy, and high reproducibility of the newly developed technology [171]. Due to the fact that this technology is considered an asset in MRD monitoring, it has recently been introduced as a molecular assay, and it has been applied in patients for the detection of mutations, such as *IDH1/2*, *DNMT3A*, and *NPM1* [172,173].

Parkin and colleagues published a work in which they tried to optimize the dPCR to characterize MRD and to be used in AML prognosis. The authors essentially applied dPCR to measure a variety of mutated alleles of recurrently mutated genes in BM samples of CR AML patients. It was possible to detect variant alleles with a frequency as low as 0.002% [174]. Koizumi et al. used mRNA to detect the presence of Wilms’ tumor 1 (*WT 1*) in several hematological malignancies, including AML, by dPCR, and compared the results with quantitative real-time polymerase chain reaction (RQ-PCR). Both methods correlated strongly with each other, but dPCR was capable of detecting lower levels of *WT1* [175]. Bussaglia et al. also applied a dPCR method for the detection of low levels of *WT1,* but, in parallel, also performed the MFC technique for immunophenotype analysis (using markers for MRD assessment) to establish some prognostic correlations [176].

The optimization of dPCR allowed the first quantitative measurement of frequent genetic mutations in AML and many other malignancies, using the detection of rare variants with high sensitivity, managing to detect 1 in 50,000 mutant cells. dPCR also allowed the provision of several conclusions on the biology of residual AML and pre-leukemia in early CR [174]. In a cohort of 99 patients the occurrence of molecular MRD (*NPM1*^mut^) by dPCR strongly correlated with RQ-PCR, showing prognostic relevance [177]. Beyond *WT1* and *NMP1*, dPCR was also performed to *IDH1/2* [115], the *PML-RARA* [178,179], *BAALC* [180], *MN1* [181].

We may conclude that dPCR is a promising tool for MRD detection since it presents good levels of sensitivity, able to achieve from 10^−4^ down to 10^−6^ [24,131]. Besides, dPCR offers other advantages, such as being much faster and easier to interpret data, without requiring technical expertise, and with lower error rates. As such, dPCR is considered a powerful and feasible tool to improve the diagnosis and stratification of patients and to monitor MRD [182].

### 6.2. LNA-qPCR

Another promising tool that has also been used is the incorporation of locked nucleic acids (LNA) nucleotides into PCR probes and primers. Briefly, LNA bases can be incorporated into any DNA or RNA oligonucleotide, inflicting a conformational modification in the local helix [183,184,185]. This alteration provides a higher hybridization affinity and stronger binding strength for complementary sequences [183,186,187] as well as better duplex formation [188]. Moreover, the incorporation of LNAs increases melting temperatures by several degrees (per LNA monomer introduced: +1–+8 °C against DNA and +2–+10 °C against RNA [189]), which permits probes and primers to be shortened [190]. All these positive aspects of the incorporation of LNAs in PCR increase the success of the amplification and, additionally, provide a higher specificity due to the elimination of undesired products [191], and, besides PCR, it has also been integrated into Molecular Beacon, TaqMan Beacon, microarray probes, and antisense oligonucleotides [189,192,193].

In the AML context, the LNAs strategy has been applied with the main goal of improving the detection of AML-specific mutations. For example, Laughlin et al. developed a method for the detection of NPM1 mutations that are frequently found in AML patients with a normal karyotype [194]. It consists in using an oligonucleotide that contains LNA nucleotides to act as a PCR clamp, thereby inhibiting amplification of the normal sequence and promoting preferential amplification of DNA with a mutation. This method provides increased analytical sensitivity of the assay [194]. In another work, the E-ice-COLD-PCR method (enhance improved and complete enrichment co-amplification at lower denaturation temperature polymerase chain reaction) was used for the detection of low levels of *NPM1* mutations [195]. This method uses a non-extendable blocker probe that integrates LNA bases to block wild-type amplification, promoting the enrichment of the four different types of *NPM1* mutations, such as A, B, D, and J [195].

Some studies have already been conducted using the LNAs for the assessment of specific mutations during MRD monitoring [114,196]. For example, the work developed by Abdelhamid and collaborators used LNA-RQ-PCR, which is a RQ-PCR-based technique incorporating LNA to modify the reverse primer in order to amplify only the mutant allele that is weakly present in the neoplastic DNA studied [196]. This strategy allowed a rapid and sensitive quantification of *IDH1* and *IDH2* gene mutations in MRD AML [196]. A similar strategy was previously used by Jeziskova et al. for the detection of IDH2 mutations [114]. In addition, a very recent work developed by Kao et al. also demonstrated the application of LNA-qPCR in detecting IDH1/2 mutations and monitoring MRD [197]. For this study, BM samples from 88 *IDH1/2*-mutated AML patients that received standard chemotherapy, azacitidine, or low-dose cytarabine as induction therapy were used. The authors then applied LNA-qPCR to quantify the *IDH1/2* mutants MRD kinetics in the samples and compared the results with those obtained for NPM1 qPCR. The results demonstrated that *IDH1/2* LNA-qPCR MRD was concordant with remission status or *NPM1*-MRD in 79.5% of patients [197]. Finally, authors demonstrated the potential of LNAs for detecting *NPM1* mutations using both PB and BM samples [198]. The results demonstrated the potential of MRD measurement by mutant *NPM1*, allowing the identification of a group at high risk of recurrence and even detection of relapse.

Thus, all these studies demonstrated the potential of LNAs integrated with quantitative PCR to provide an easier but more specific and sensitive way to detect low levels of specific and relevant mutations in AML and MRD conditions.

### 6.3. Surface-Enhanced Raman Scattering for Diagnosis of AML

At present, Raman spectroscopy and its variant surface-enhanced Raman scattering (SERS) spectroscopy are among the most powerful analytical techniques available for the analysis of biochemical markers. Its application has already been demonstrated in a wide number of fields, from environmental pollution [199], food industry [200], biomedical [201], medicine [202,203] to arts [204]. Raman spectroscopy is a scattering technique that studies the specific vibrations of molecules [205] by the analysis of their characteristic Raman spectrum (Raman intensity versus wavelength shift spectrum) [206]. This technique provides a vibrational fingerprint of the studied sample that can be directly correlated to its structure, constituents, symmetry, and environment [207]. Thus, Raman spectroscopy can be used to determine chemical composition, molecular structures/conformations, and interactions between different molecules, allowing qualitative and quantitative results [207]. Qualitative analysis can be performed by measuring the frequency of scattered radiation, while quantitative analysis is performed by measuring the intensity of scattered radiation [208]. However, the sensitivity of conventional Raman spectroscopy is intrinsically low, which limits its wide application in the biomedical field [207]. One way to overcome this limitation is to use metal nanoparticles or other plasmonic nanostructures to enhance the Raman signal of molecules that are in their vicinity, known as SERS [209].

SERS is an advanced analytical technique that can be used for the ultra-sensitive detection of various analytes, with astonishingly low detection limits, even up to a single molecular level [210]. This technology was already explored in rare cells detection in the distinction of different cell populations or even in DNA mutation detection. For example, Nima et al. demonstrated a strategy based on SERS to detect CTCs, where four different antibodies and Raman tags were used, showing the possibility of identifying a single cancer cell, with high accuracy/precision, in the middle of millions of blood cells [211]. Wu et al. also developed three types of active SERS nanoparticles to detect CTCs in blood samples with high sensitivity (detection limit of 1 cell/mL) and specificity, without prior enrichment [212]. Additionally, an efficient and non-invasive method was also developed based on an active SERS platform. Briefly, this method consists of the incorporation of the platform in the microfluidic device to isolate CTCs, promoting a label-free detection and its identification through structural and biochemical analysis [213]. This demonstrates the relevance and applicability of SERS in the difficult task of detecting rare cells.

On the distinction of different cell populations, Teixeira et al. demonstrated the potential of the SERS technique to detect the profiles of different cell types (malignant and non-malignant) and to distinguish them based on spectral differences [214]. In a recent study, Oliveira et al. demonstrated the multiplex phenotyping of single cancer cells using SERS probes [215]. Furthermore, the possibility to detect DNA mutations using SERS was also demonstrated. More precisely, a SERS chip with the ability to detect specific KRAS mutations and differentiate between wild-type and mutated KRAS genes in different cancer cells was developed [216]. The same SERS chip was optimized and combined with supervised machine learning allowing the intelligent cancer classification according to its mutational landscape [217]. The application of this sensitive and precise technology, with added value in the detection of malignant cells, has already been explored in the context of leukemia or even to distinguish these cells from the healthy ones. In 2014, González-Solís and colleagues mentioned the relevance of SERS technology associated with leukemia. They analyzed the biochemistry of blood serum from leukemia patients and healthy donors, through Raman spectroscopy, and managed to distinguish between normal and leukemia cells, and also to identify different types of leukemia [218]. In CLL, a SERS technique was used to detect leukemic cells, using SERS probes composed by nanoparticles functionalized with PEG and antibodies (to recognize three surface proteins of interest in malignant B cells). To confirm the results of this method, flow cytometry was used [219].

In ALL, the differentiation between normal B-lymphocytes and B-ALL cells at different maturation stages using Raman spectroscopy and Principal component analysis (PCA) was demonstrated, with important implications for the clinical practice. The high sensitivity of Raman and feature-rich Raman spectra offers the possibility of multiplex detection, and it could represent a major step in blood analysis through the realization of a non-destructive, label-free Raman-based flow cytometer [220]. Furthermore, in the context of B-cell hematological malignancy, a SERS-based strategy was also used to simultaneously detect two MRD surface markers (CD19 and CD20) in patient blood samples. The results were compared with flow cytometry and demonstrated the great potential of the approach for MRD detection with high sensitivity [221].

Finally, in the context of AML, a preliminary study was carried out using a sensitive and robust platform, based on nanoparticles and hollow core photonic crystal fibers, to monitor and detect leukemic cells (using HL-60 cells) [222]. In addition, electroporation-based SERS spectroscopy using silver nanoparticles was used to detect AML cells (HL-60 and K562 cells) and also differentiate them from healthy cells (PBMCs, Peripheral blood mononuclear cells) [223]. It is important to note that some SERS methods have been also used in AML patient samples, more precisely in APL subtype, to discriminate subtypes or molecular tests. For example, Ye et al. used Ag nanoparticles-based SERS technique to discriminate acute monocytic leukemia and the AML subtype acute promyelocytic leukemia using plasma from patient samples [224]. Another study also demonstrated the possibility of using SERS (through Ag nanoparticles) to distinguish between AML subtypes and control samples, and also for the prognostic stratification of AML patients using characteristic SERS bands [225]. Interestingly, for the discrimination between AML patients and control samples, the relevant specific patterns referred to nucleic acids, and also amino acids, glucose (and products), and protein (and derivatives). On the other hand, to distinguish between different subtypes of AML, different nucleic acids patterns were used. This study demonstrated the potential of SERS technique to be translated to clinical practice, then, and to aid in the differential diagnostic and prognostic stratification of AML patients [225].

A different approach developed by Moisoiu et al. showed the application of SERS for the detection of cancer DNA, more precisely for the characteristic methylation pattern of cancer DNA without pre-amplification in AML patients. The results demonstrated that with SERS, it is possible to detect a unique methylation pattern of cancer DNA and, even more, to promote a discrimination between control samples and AML patients, with a specificity of 82% and a sensitivity of 82% [226].

The studies described above have evidenced the high sensitivity and precision of Raman spectroscopy, and its capacity to differentiate tumor cells from healthy blood cells in a non-destructive way, and even to discriminate patient subtypes. Despite the very promising potential of this technique in cancer and also specifically in AML context, more studies are needed. For example, no specific studies using SERS for the detection of CLCs from blood samples and for the evaluation of MRD or even for specific DNA mutations in AML have been described so far.

## 7. Conclusions

The detection of MRD has a strong clinical relevance, but it is still the greatest challenge in AML disease management. New technological advances have been focused on increasing sensitivity and specificity to create essential tools for the early diagnosis of AML and its relapse. Some of the most promising tools to improve the current landscape include microfluidics and SERS or a combination of both. Indeed, several techniques may be needed, in order to maximize the availability of clinically useful information, with the ultimate goal of promoting more personalized treatment approaches.

## Figures and Tables

**Table 1:** Summary of the classification of AML with percentage of blasts required for the diagnosis [24,39].

AML with Recurrent Genetic Abnormalities (Now Requiring ≥10% Blasts in BM or PB)
APL with a translocation between chromosomes 15 and 17-t(15;17)(q22;q12)/*PML-RARA*
APL with other *RARA* rearrangements
AML with a translocation between chromosomes 8 and 21-t(8;21)(q22;q22); *RUNX1::RUNX1T1*
AML with a inversion or translocation in chromosome 16-inv(16)(pl3.1q22) or t(16;16)(p13.1;q22)/*CBFB::MYH11*
AML with a translocation between chromosomes 9 and 11-t(9;11)(p21.3;q23.3); *MLLT3::KMT2A*
AML with other *KMT2A* rearrangements
AML with a translocation between chromosomes 6 and 9-t(6;9)(p22.3;q34.1)/*DEK-NUP214* AML with a translocation or inversion in chromosome 3-inv(3)(q21.3q26.2) or t(3;3)(q21.3;q26.2)/*GATA2;MECOM(EVI1)*
AML with other *MECOM* rearrangements
AML with other rare recurring translocations
AML with mutated *NPM1*
AML with in-frame bZIP *CEBPA* mutations
AML with translocation between chromosomes 9 and 22 t(9;22)(q34.1;q11.2)/*BCR::ABL1*
**Myeloid Sarcoma**
**Acute leukemia of ambiguous lineage**
Acute undifferenciated leukemia Mixed phenotype acute leukemia (MPAL) with translocation between chromosomes 9 and 22-t(9;22)(q34.1;q11.2)/*BCR::ABL1*
MPAL t(v;11q23.3)/*KMT2A*-rearranged
MPAL, B/myeloid, not otherwise specified
MPAL, T/myeloid, not otherwise specified
**Categories designated AML** (≥20% blasts in BM and PB) **or MDS/AML** (if ≥10 to 19% blasts in BM and PB)
AML with mutated *TP53*
AML with myelodysplasia-related gene mutations defined by: mutations in *ASXL1, BCOR, EZH2, RUNX1, SF3B1, SRSF2, STAG2, U2AF1,* and/or *ZRSR2*
AML with myelodysplasia-related cytogenetic abnormalities^j^
AML not otherwise specified
**Myeloid neoplasms associated to Down syndrome**
Transient abnormal myelopoiesis associated with Down syndrome
Myeloid leukemia associated with Down syndrome
**Blastic plasmacytoid dendritic cell neoplasm**

The classification is adapted from references [24,39].

## Data Availability

The data can be shared up on request.
